# Antagonistic effects of endophytic fungi from *Camellia reticulata* pedicels on yeasts: implications for antimicrobial mechanism of nectar

**DOI:** 10.3389/fpls.2024.1494855

**Published:** 2024-11-15

**Authors:** Rong Huang, Qingxin Meng, Lijie Xun, Xiaoman Wu, Dan Yue, Wenzheng Zhao, Xia Dong, Xueyang Gong, Kun Dong

**Affiliations:** ^1^ Yunnan Provincial Engineering and Research Center for Sustainable Utilization of Honey Bee Resources, Eastern Bee Research Institute, College of Animal Science and Technology, Yunnan Agricultural University, Kunming, China; ^2^ Institute of Sericulture and Apiculture, Yunnan Academy of Agricultural Sciences, Mengzi, China; ^3^ College of Food Science and Technology, Yunnan Agricultural University, Kunming, China

**Keywords:** *Camellia reticulata*, endophytic fungi, nectar yeasts, antagonistic activity, antimicrobial mechanism, *Alternaria alternata*

## Abstract

Endophytic fungi are extensive in plant tissues and involved in the defense against stress from harmful microbes. The interaction between pedicel endophytic fungi and nectar yeasts is critical for maintaining nectar homeostasis. This study used *Camellia reticulata* as the research subject. High-throughput sequencing revealed that the community composition of endophytic fungi in the pedicel is dominated by Ascomycota and Basidiomycota. Their abundance varies at different taxonomic levels, showing sample variability. In total, 27 endophytic fungal isolates were isolated and screened from the pedicel under laboratory conditions. They exhibited antagonistic effects against three nectar yeasts (*Metschnikowia reukaufii*, *Cryptococcus laurentii*, and *Rhodotorula glutinis*) and displayed morphological and physiological diversity. The isolates were classified into the phylum Ascomycota and further categorized into the genera *Alternaria*, *Trichoderma*, *Fusarium*, and *Dactylaria*. The endophytic fungus D23, which effectively antagonizes nectar yeasts, was identified as *Alternaria alternata*. This fungus produces various secondary metabolites, including antibiotics such as penicillin G, grandiomycin, and cephalosporin C. The metabolic pathways involved include the biosynthesis of plant secondary metabolites, phenylpropanoids, amino acids, nucleotides, and antibiotics. The endophytic fungal community in *C. reticulata* pedicel is rich and diverse, making it a valuable material for screening antagonistic strains. This study provides a theoretical basis for the antagonistic effects of endophytic fungal metabolites from the pedicel of *C. reticulata* against nectar yeasts, highlighting their significance in maintaining nectar stability and reproductive fitness in cross-pollinated plants.

## 1 Introduction

Nectar is an energy reward from plants for pollinators. Yeast commonly inhabits the nectar of most plants. Increased pollinator visits enhance yeast density in the nectar. This increase in density and subsequent metabolic activity alters the chemical composition of the nectar, particularly affecting sugar and amino acid levels (Canto and Herrera, 2012; Peay et al., 2012). However, such changes may weaken the nectar’s appeal to specific pollinators and reduce visitation rates (Canto and Herrera, 2012; Schaeffer et al., 2017). Yeasts in plant nectar, primarily classified under Ascomycota and Basidiomycota, include genera such as *Metschnikowia*, *Cryptococcus*, *Rhodotorula*, *Saccharomyces*, and *Sporobolomyces* (Herrera et al., 2009; Pozo et al., 2012; Jacquemyn et al., 2013). Without effective antimicrobial mechanisms, nutrient-rich nectar likely supports high microorganism proliferation, diminishing its value for plant reproduction. Researchers have investigated the chemical components and defensive functions of nectar worldwide. They found that factors like pH, high osmotic pressure, and various secondary metabolites or proteins contribute to the antimicrobial properties of the nectar (Pusey, 1999; Brysch-Herzberg, 2004; Herrera et al., 2011; Álvarez-Pérez et al., 2013). However, research into the antimicrobial substances in nectar and their functions remains exploratory, and the role of endophytes is also under examination.

Endophytic fungi are crucial for the plant microenvironment, establishing a dynamic balance through colonization and operational mechanisms. They form communities with significant ecological functions (Philippot et al., 2013). These fungi enhance plant stress resistance, promote growth (Compant et al., 2005; Zhang et al., 2006), and suppress pathogen populations by spatial localization. They produce various antimicrobial metabolites that help plants resist microbial stress (Zhang et al., 2006; Pieterse et al., 2014). For instance, Stinson et al. (2003) isolated *Gliocladium* sp. from *Eucryphia cordifolia*. This fungus produces volatile organic compounds, such as cyclooctatetraene and 3-methyl-1-butanol, with strong antimicrobial effects against plant pathogens like *Pythium ultimum* and *Verticillium dahliae*. Richardson et al. (2015) isolated two Massarinaceae species from *Pinus strobus*, producing phenolic compounds that inhibit *Bacillus subtilis* and *Microbotryum violaceum*. Hiremani et al. (2020) isolated two species of *Nigrospora sphaerica* from *Gossypium arboreum*, producing volatile organic compounds effective against *Corynespora cassiicola*. Recent research has found that endophyte colonization can alter the composition of volatile metabolites in flowers, attract more pollinators, and enhance the reproductive adaptability of plants, providing new insights into the resistance mechanism of endophyte-assisted nectar against yeast (González-Mas et al., 2023). However, studies on the antagonistic effects of endophytic fungi against nectar yeasts are rare.

*Camellia reticulata* has been cultivated in Yunnan for a long time and is a rare plant protected in China (Feng, 1980). Its seed-extracted oil is rich in unsaturated fatty acids and antioxidants and has nutritional value comparable to olive oil, which makes it a geographical indication product in China (Qin et al., 2023). Due to self-incompatibility, *C. reticulata* heavily depends on pollinators for fruit set and seed production. The plant has a long flowering period, produces abundant nectar, and faces significant microbial contamination during pollination. Preliminary studies in our laboratory found that nectar from flowers blooming for many days maintains low microbial concentration. However, yeast abundance significantly increases during *ex vivo* fermentation, deteriorating nectar’s physicochemical properties (unpublished data). Importantly, the antimicrobial mechanism within *C. reticulata* nectar remains unclear. The bacteriostatic effects of endophytic fungi colonizing plant tissues may provide new explanations for their antimicrobial mechanisms. Pedicels, which are plant tissues connecting to flowers, play a crucial role in spatial distribution and serve as important conduits for nutrient supply (Zhou, 2015). The endophytic fungi colonize the pedicel near the flowers for extended periods, making them valuable microbial resources that can antagonize nectar yeasts in an auxiliary role. However, there is limited understanding of the community composition and functions of endophytic fungi colonizing *C. reticulata* pedicels. Accordingly, this study focuses on *C. reticulata* pedicels to analyze its endophytic fungal communities, screen isolates inhibiting nectar yeast, and analyze their metabolic products. Specific research objectives include (1) high-throughput sequencing to analyze endophytic fungi diversity in *C. reticulata* pedicels; (2) isolation of endophytic fungi from pedicels; (3) screening of endophytic fungi with antagonistic activity against nectar yeasts and evaluating these isolates’ inhibitory effects; (4) morphological and molecular biological identification of these isolates; (5) evaluation of minimum inhibitory concentration (MIC) and antibacterial potential of ethyl acetate extract from efficient isolate D23; and (6) non-targeted metabolomics analysis of metabolic products and antimicrobial substances from efficient isolate D23. This study analyzed endophytic fungi diversity in *C. reticulata* pedicels and screened for fungi antagonizing nectar yeast. Our results provide insights into mechanisms by which cross-pollinated plants resist yeast stress, regulate nectar stability, and maintain reproductive fitness.

## 2 Materials and methods

### 2.1 Sampling information and plant sample collection

In January and February 2024, *C. reticulata* plant samples were collected from Shaba Forestry Farm (98°5’86’’ E, 24°9’57’’ N) in Tengchong, Yunnan Province. This site has a tropical monsoon climate, with an average annual temperature of 14.6°C, approximately 2,167 h of sunshine per year, an average annual precipitation of 1,531 mm, and an average altitude of 2,149 m. During sampling, healthy *C. reticulata* plants were selected. Flower buds and the lower 6 cm of stems were cut using sterilized scissors (sterilized with 75% alcohol) as plant samples. These samples were placed in sterile bags and transported to the laboratory at 4°C for further processing.

### 2.2 Sample surface sterilization

Plant samples were washed in flowing tap water. Under sterile conditions, flower buds and stems were trimmed to leave only pedicels. Following the method of Cardoso et al. (2020), pedicels were subjected to stringent surface sterilization. They were first rinsed with sterile distilled water and then immersed in 75% ethanol for 1 min. Pedicels were washed four times with sterile distilled water and dried with sterile filter paper. Next, they were soaked in 5% sodium hypochlorite solution for 2 min, rinsed four times with sterile distilled water, and dried again with sterile filter paper. To verify sterilization effectiveness, 100 µL of the sterile water from the final wash was plated on Luria broth (LB) and tryptic soy agar (TSA) media and incubated at 30°C for 72 h. The absence of microorganism growth confirmed that sterilization was successful and that pedicels were suitable for subsequent endophytic fungal screening and identification.

### 2.3 Diversity analysis of endophytic fungi

Surface-sterilized pedicels were ground in liquid nitrogen, and total DNA from the pedicel microbiota was extracted using the E.Z.N.A.^®^ Soil DNA Kit (Omega Bio-tek, Norcross, GA, U.S.). DNA concentration and purity were measured with a NanoDrop 2000 machine, and DNA quality was assessed using 1% agarose gel electrophoresis. PCR amplification of the ITS1-ITS2 region of genomic DNA was performed using endophytic fungal primers ITS1F (5’-CTTGGTCATTTAGAGGAAGTAA-3’) and ITS2R (5’-GCTGCGTTCTTCATCGATGC-3’) (Gardes and Bruns, 1993). PCR products were recovered using the AxyPrep DNA Gel Recovery Kit, and the target fragment size was confirmed with 2% agarose gel electrophoresis. Quantification was conducted using the QuantiFluor™-ST blue fluorescence quantification system. Purified PCR products were sent to Shanghai Majorbio Biomedical Technology Co., Ltd. for Illumina high-throughput sequencing. Sequences from raw data were optimized, and OTU clustering analysis of non-redundant sequences was performed using the Usearch software platform (similarity: 97%). Diversity analysis was conducted using the I-Sanger bioinformatics cloud platform.

### 2.4 Isolation, purification, and preservation of endophytic fungi

Sterilized pedicels were cut into small pieces using a sterile scalpel and transferred to the potato dextrose agar (PDA) medium. They were incubated at 28°C in an incubator for 3-7 days. Once fungal mycelium grew at the edges of the plant tissue, hyphae were isolated from the medium based on colony characteristics using a scalpel and transferred to a fresh PDA medium for purification. After several rounds of purification to achieve uniform colony morphology, colonies were transferred to a PDA slant and stored at 4°C for preservation, with subculturing every 30 days to prevent isolate degradation. Additionally, surface mycelium was scraped using an inoculation spatula, placed in a 50% glycerol cryopreservation tube, mixed thoroughly, and frozen at -80°C for long-term storage.

### 2.5 Screening of isolates antagonizing nectar yeasts

Three types of nectar yeasts provided by the China General Microbiological Culture Collection Center were used for testing: *Metschnikowia reukaufii*, *Cryptococcus laurentii*, and *Rhodotorula glutinis*. The yeast strains were first revived using the malt extract broth (MEB) medium. After 24 h, 1 mL of each strain suspension was taken and diluted with sterile phosphate buffered saline (PBS) to a 0.5 McFarland standard (approximately 10^8^ CFU/mL). The prepared yeast suspension was evenly spread onto a PDA medium using a sterile cotton swab for testing. Additionally, 5 mm diameter agar discs medium covered with fungal mycelium were punched from the PDA medium. After overnight incubation at 28°C, isolates exhibiting inhibition zones were initially selected. The endophytic fungi initially screened with antagonistic activity against nectar yeast were then inoculated into potato dextrose broth (PDB) medium and cultured at 135 rpm and 28°C for 5 days in a shaking incubator. Afterward, each endophytic fungal culture (10 mL) was centrifuged at 12,000 rpm for 15 min to obtain the supernatant, which was then filtered through a 0.22 μm membrane to produce a sterile fermentation extract for each isolate. Oxford cups with a diameter of 5 mm were placed on the medium surface, and 100 μL of the fermentation extract was added to each cup. PDB medium served as the blank control (CK), and Amphotericin B was used as the positive control (+). All media were wrapped with sealing film and incubated at 28°C for 24 h. Finally, the inhibition zone diameters for each isolate were measured.

### 2.6 Identification of endophytic fungi

Firstly, morphological characteristics of endophytic fungi exhibiting antagonistic activity against nectar yeast were recorded, including colony color, size, surface features, and pigment production. DNA was extracted from selected endophytic fungi using the E.Z.N.A.^®^ Soil DNA Kit, following the manufacturer’s instructions. PCR amplification was performed with fungal primers ITS1 (5’-TCCGTAGGTGAACCTGCGG-3’) and ITS4 (5’-TCCTCCGCTTATTGAATGC-3’) (White et al., 1990). Amplification results were analyzed by 1% agarose gel electrophoresis (150V/100mA for 20 min). Target DNA fragments were recovered using a QIAquick agarose gel extraction kit. The purified PCR products were sequenced using the ABI3730XL system. Sequencing data were compared to the NCBI database using BLAST. The accession number and percent identity for the strain that showed the highest alignment score with the isolate were obtained.

### 2.7 Analysis of isolate metabolites showing effective antagonism against nectar yeasts

#### 2.7.1 Extraction of secondary metabolites of isolate

First, the endophytic fungus D23 was activated and rejuvenated. The fungus was transferred to PDA medium and incubated at 28°C for 4 days. After spores and mycelium matured, 5 mm mycelial discs were prepared and inoculated into a PDB medium. The culture was incubated at 28°C and 135 rpm for 3 days to obtain a fungal liquid culture. Next, 200 g of rice and 250 mL of MEB medium were added to each 2L wide-mouthed bottle. After autoclaving and cooling, 100 mL of fungal liquid culture was inoculated, mixed well, and incubated at 28°C for 30 days. Once the rice was fully colonized by mycelium, it was transferred to a sterilized tray, dried completely at 45°C, and then crushed. To extract the compounds, 100 g of the powdered rice was mixed with 500 mL of ethyl acetate. The mixture was extracted in a 45°C water bath for 3 h, then filtered, and the organic phase was collected. The extraction was repeated with ethyl acetate for each batch of powder a total of three times. The combined organic phases were transferred to a rotary evaporator to evaporate the solvent at 45°C. After evaporation, the organic solvent was recovered, and the crude extract was dissolved in methanol, dried in a ventilated cabinet, and stored at 4°C for future use.

#### 2.7.2 MIC estimation of isolate metabolites against nectar yeasts

MIC values were determined using the broth dilution method following Clinical and Laboratory Standards Institute (CLSI) guidelines, with Amphotericin B as the positive control (Wang et al., 2023). In the first row of a 96-well plate, 180 µL of yeast suspension (1.25 × 10^6^ CFU/mL) was added, while 100 µL was added to the remaining wells. A crude extract solution of D23 in DMSO (10 mg/mL) was prepared, and 20 µL was added to the first row of the wells. A two-fold serial dilution was then performed across the plate, and turbidity in each well was observed to determine the lowest D23 concentration required to inhibit yeast growth.

#### 2.7.3 LC-MS/MS analysis of metabolites composition in the isolate

Non-targeted metabolomics LC-MS/MS was conducted to analyze active components in the ethyl acetate extract of D23. The MSConvert tool in the Proteowizard software package (v3.0.8789) was used to convert the original mass spectrometry downloaded file into mzXML format. Peak detection, filtering, and alignment were performed using the R XCMS package (v3.12.0), resulting in a quantitative metabolite list. Systematic errors were corrected using support vector regression based on QC samples, and substances with relative standard deviation (RSD) > 30% in QC samples were filtered out. Substance identification was conducted using public databases such as HMDB, MassBank, LipidMaps, mzCloud, and KEGG, with parameters set to < 30 ppm. Metabolite identification was conducted at Shanghai Majorbio Biomedical Technology Co., Ltd.

## 3 Results

### 3.1 Community structure of endophytic fungi in the pedicel of *C. reticulata*

#### 3.1.1 OTU analysis of endophytic fungi in pedicel of *C. reticulata*

After the final rinse of the pedicel, plating on LB and TSA media followed by 72 h of incubation revealed no colony growth, indicating successful tissue sterilization. Consequently, the isolated fungi were derived from the endophytic colonization of the pedicel and not from the environment.

**Figure 1:**
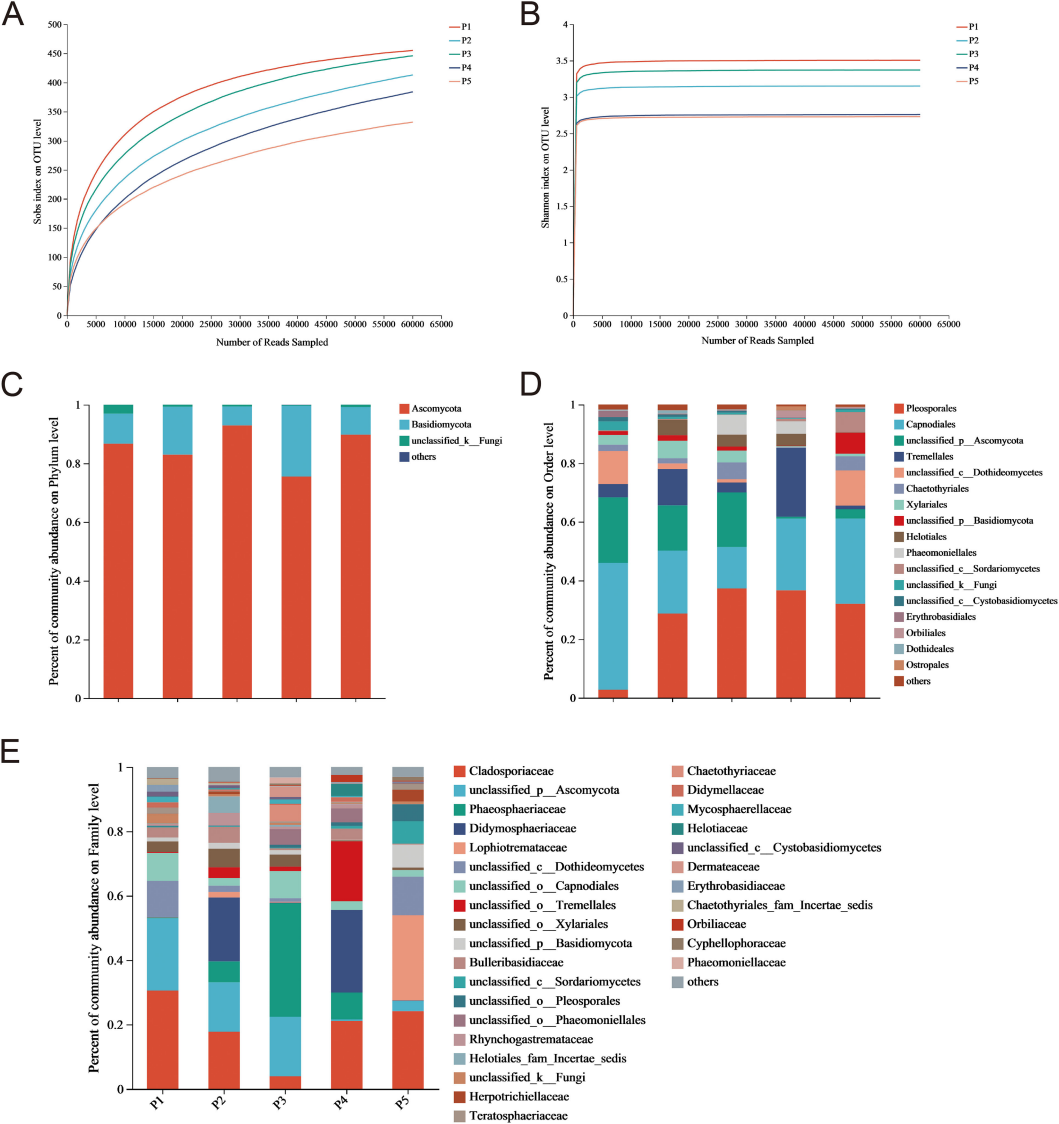
Rarefaction curves for **(A)** Sobs and **(B)** Shannon Indices, and relative abundance of endophytic fungi in *C. reticulata* pedicels at the **(C)** phylum, **(D)** order, and **(E)** family levels.

OTUs of endophytic fungi from the pedicel were classified based on 97% similarity, resulting in an average of 72, 061 effective sequences with a mean length of 238 bp. Sequence lengths primarily ranged between 201 and 280 bp, aligning with expected amplification lengths and demonstrating good concentration (**Figures 1A, B**). A total of 1,034 OTUs was obtained, distributed across 4 phyla, 24 classes, 77 orders, 161 families, and 267 genera. This indicates that the endophytic fungi in the pedicel of *C. reticulata* exhibit rich community diversity at various taxonomic levels.

#### 3.1.2 Abundance analysis of endophytic fungi in *C. reticulata* pedicel

The endophytic fungal communities detected in *C. reticulata* pedicels included Ascomycota, Basidiomycota, and unclassified_k_Fungi (**Figure 1C**). Ascomycota and Basidiomycota together accounted for 90% of the abundance. The proportion of Ascomycetes in each group reached 60%, indicating it is a dominant phylum. These endophytic fungi were distributed across 15 orders, with varying proportions and strain uniformity in each sample. Pleosporales, Capnodiales, unclassified_p_Ascomycota, Tremellales, unclassified_c_Dothideomycetes, Xylariales, and Chaetothyriales were the dominant orders, representing over 80% of each sample (**Figure 1D**). These fungi were also distributed across 30 families, but dominant families differed among samples (**Figure 1E**). In some samples, dominant families accounted for over 20%, including Cladosporiaceae, unclassified_p_Ascomycota, Phaeosphaeriaceae, Didymosphaeriaceae, and Lophiotremataceae. However, only some endophytic fungi were classified to the family level, with others classified only to the order, class, phylum, or kingdom level. This suggests that *C. reticulata* pedicels harbor a diverse range of fungal species, warranting further exploration.

### 3.2 Screening of endophytic fungi antagonizing nectar yeasts in *C. reticulata* pedicels

After rigorous surface sterilization, 118 endophytic fungal isolates were isolated from *C. reticulata* pedicels based on different colony characteristics. Of these, 27 isolates were preliminarily screened for antagonistic activity against the three nectar yeasts, indicated by inhibition zones. Among these, 20 isolates were inhibitory to *M. reukaufii*, 25 to *C. laurentii*, and 22 to *R. glutinis*. Isolates B1, B2, B10, B18, B27, D14, D23, E23, E52, E53, E57, E78, E79, and E80 exhibited antagonistic activity against all three nectar yeasts (**Table 1**).

**Table 1:** Preliminary screening results of endophytic fungi with antagonistic activity (indicated by inhibition zone) against nectar yeasts in *C. reticulata* pedicels.

Isolates	Nectar yeasts		
	*Metschnikowia reukaufii*	*Cryptococcus laurentii*	*Rhodotorula glutinis*
B1	+	+	+
B2	+	+	+
B6	–	+	+
B10	+	+	+
B18	+	+	+
B24	+	+	–
B26	+	+	–
B27	+	+	+
D9	–	+	+
D14	+	+	+
D15	–	+	+
D23	+	+	+
E17	+	+	–
E23	+	+	+
E31	+	+	–
E35	+	+	–
E37	–	+	+
E40	–	+	+
E46	–	+	+
E52	+	+	+
E53	+	+	+
E57	+	+	+
E61	–	–	+
E69	+	–	+
E78	+	+	+
E79	+	+	+
E80	+	+	+

In addition, endophytic fungi with antagonistic activity against nectar yeast were again screened using fermentation substances. Our results showed that fermentation substances from isolates with preliminary antagonistic activity also exhibited antagonistic activity against yeasts (**Table 2**). Inhibition zone measurements revealed that endophytic fungus D23 had the strongest inhibitory effect on all three yeasts, with inhibition zone diameters of 16.37 ± 0.09 mm for *M. reukaufii*, 15.03 ± 0.65 mm for *C. laurentii*, and 13.97 ± 0.46 mm for *R. glutinis*.

**Table 2:** Inhibition zone diameters of endophytic fungi with antagonistic activity against nectar yeasts (mean ± S.E., unit: mm).

Isolates	Inhibition zone diameter (mm)		
	*Metschnikowia reukaufii*	*Cryptococcus laurentii*	*Rhodotorula glutinis*
B1	7.27 ± 0.41	6.90 ± 0.29	9.20 ± 0.28
B2	10.83 ± 0.24	10.43 ± 0.63	9.37 ± 0.33
B6	–	5.47 ± 0.05	6.50 ± 0.08
B10	7.97 ± 0.17	8.80 ± 0.42	8.37 ± 0.12
B18	–	–	6.47 ± 0.37
B24	8.00 ± 0.14	7.73 ± 0.09	–
B26	7.73 ± 0.39	7.87 ± 0.74	–
B27	8.13 ± 0.17	8.07 ± 0.48	8.13 ± 0.29
D9	–	8.33 ± 0.12	8.63 ± 0.12
D14	8.30 ± 0.37	7.93 ± 0.25	8.90 ± 0.29
D15	–	9.30 ± 0.14	8.27 ± 0.25
D23	16.37 ± 0.09	15.03 ± 0.65	13.97 ± 0.46
E17	9.30 ± 0.29	9.27 ± 0.17	–
E23	–	8.90 ± 0.37	8.17 ± 0.09
E31	6.83 ± 0.29	10.87 ± 0.29	–
E35	9.00 ± 0.16	8.20 ± 0.29	–
E37	–	12.10 ± 0.37	8.40 ± 0.16
E40	–	12.37 ± 0.45	8.17 ± 0.25
E46	–	11.47 ± 0.47	11.33 ± 0.54
E52	10.83 ± 0.25	10.33 ± 0.41	11.17 ± 0.54
E53	8.87 ± 0.25	7.97 ± 0.34	8.23 ± 0.31
E57	6.77 ± 0.21	8.13 ± 0.21	9.77 ± 0.52
E61	–	–	8.40 ± 0.08
E69	7.83 ± 0.25	–	7.93 ± 0.12
E78	11.13 ± 0.25	9.23 ± 0.42	11.10 ± 0.50
E79	9.17 ± 0.21	7.80 ± 0.16	9.27 ± 0.26
E80	9.77 ± 0.52	9.00 ± 0.16	11.37 ± 0.54
Amphotericin B	24.30 ± 3.48	20.27 ± 0.88	21.73 ± 0.34

### 3.3 Identification of endophytic fungi antagonizing nectar yeasts

#### 3.3.1 Morphological characterization of the isolates

**Figure 2:**
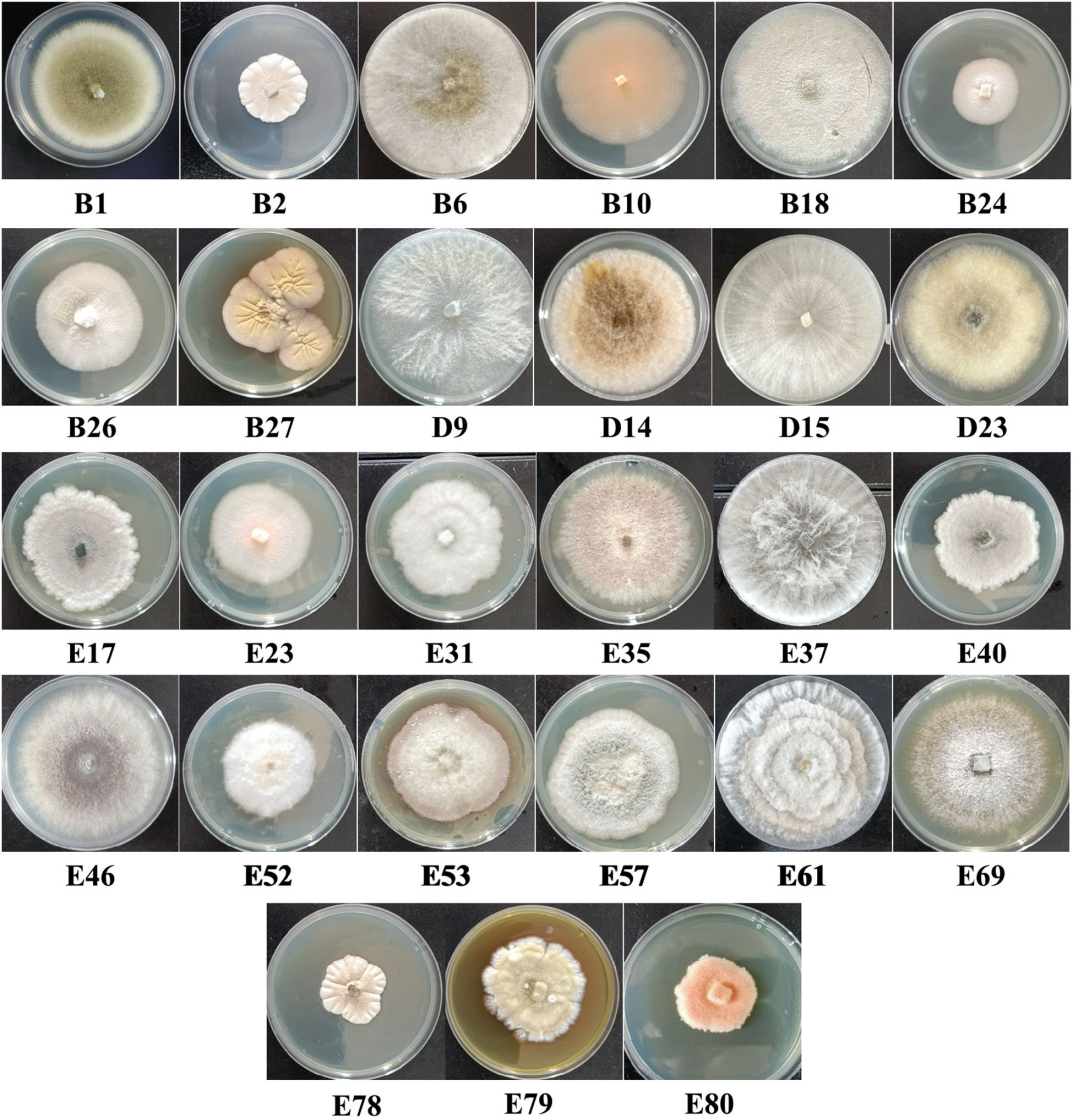
Colony morphology of endophytic fungi within the *C. reticulata* pedicel under cultivation conditions of 28°C on PDA medium.

Isolates isolated from the *C. reticulata* pedicel showed diverse colony features, including color and hyphal characteristics. In early growth stages, colonies were mainly white or gray, with some light yellow or light pink. As colonies grew, colors gradually changed to gray-green, gray-brown, black, or orange. Some produced pigments altering the medium’s color to gray-brown, gray-yellow, light yellow, brown, reddish-brown, brown-yellow, and black (**Figure 2**).

#### 3.3.2 Molecular identification of isolates

Endophytic fungi isolated from the *C. reticulata* pedicel were all Ascomycota, classified into the Eurotiomycetes, Sordariomycetes, and Dothideomycetes classes. They were further categorized into genera such as *Trichoderma*, *Fusarium*, *Daldinia*, *Nodulisporium*, *Hypoxylon*, *Colletotrichum*, *Coniochaeta*, *Epicoccum*, *Alternaria*, *Endomelanconiopsis*, and *Botryosphaeria* (**Table 3**). Among them, fungus D23, with efficient antagonism against nectar yeast, was identified as *Alternaria alternata*.

**Table 3:** Molecular identification of endophytic fungi in *C. reticulata* pedicel.

Isolates	Species	Length (bp)	Closest number	ldentity (%)
B1	*Aspergillus niger*	575	PQ041979.1	99.83
B2	*Penicillium citrinum*	533	OK103925.1	100
B6	*Trichoderma atroviride*	602	LC785690.1	100
B10	*Coniochaeta* sp.	535	JX838853.1	99
B18	*Hypoxylon griseobrunneum*	524	OW985412.1	99.61
B24	*Phaeoacremonium scolyti*	581	KC166687.1	99.65
B26	*Phaeoacremonium scolyti*	581	KC166687.1	99.65
B27	*Penicillium implicatum*	571	MF687276.1	100
D9	*Daldinia bambusicola*	544	KU940155.1	99.81
D14	*Alternaria tenuissima*	549	MZ047512.1	100
D15	*Trichoderma harzianum*	598	MF109019.1	99.83
D23	*Alternaria alternata*	550	OQ727506.1	100
E17	*Daldinia eschscholtzii*	553	MZ724905.1	99.82
E23	*Colletotrichum citri*	577	MZ724781.1	99.82
E31	*Trichoderma gamsii*	592	PP469586.1	99.66
E35	*Colletotrichum fructicola*	558	OQ511318.1	99.82
E37	*Botryosphaeria dothidea*	994	LC317472.1	99.35
E40	*Daldinia eschscholtzii*	554	KJ466979.1	99.82
E46	*Colletotrichum siamense*	557	MZ066745.	99.82
E52	*Fusarium acuminatum*	542	MK583544.1	99.45
E53	*Alternaria solani*	548	KX452728.1	99.82
E57	*Endomelanconiopsis microspora*	566	MH862651.1	99.46
E61	*Nodulisporium* sp.	685	EF694672.1	98.93
E69	*Colletotrichum camelliae*	556	ON025203.1	99.28
E78	*Penicillium citrinum*	539	KY921947.1	99.81
E79	*Epicoccum nigrum*	527	OP696974.1	99.81
E80	*Fusarium acuminatum*	557	MZ724839.1	99.27

### 3.4 Antagonistic activity of *A. alternata* against nectar yeasts

#### 3.4.1 Determination of *A. alternata* MIC against nectar yeasts

The MIC of *A. alternata* was below 500 µg/mL for all three tested nectar yeasts, showing significant potential to inhibit them, similar to Amphotericin B. Specifically, *A. alternata* exhibited the highest antimicrobial activity against *M. reukaufii*, with a MIC of 125 µg/mL, followed by a MIC of 250 µg/mL against *C. laurentii* and 500 µg/mL against *R. glutinis*. For Amphotericin B, MIC values for the three nectar yeasts were 1.95, 3.91, and 3.91 µg/mL, respectively.

#### 3.4.2 Compositional analysis of the *A. alternata* metabolites

**Figure 3:**
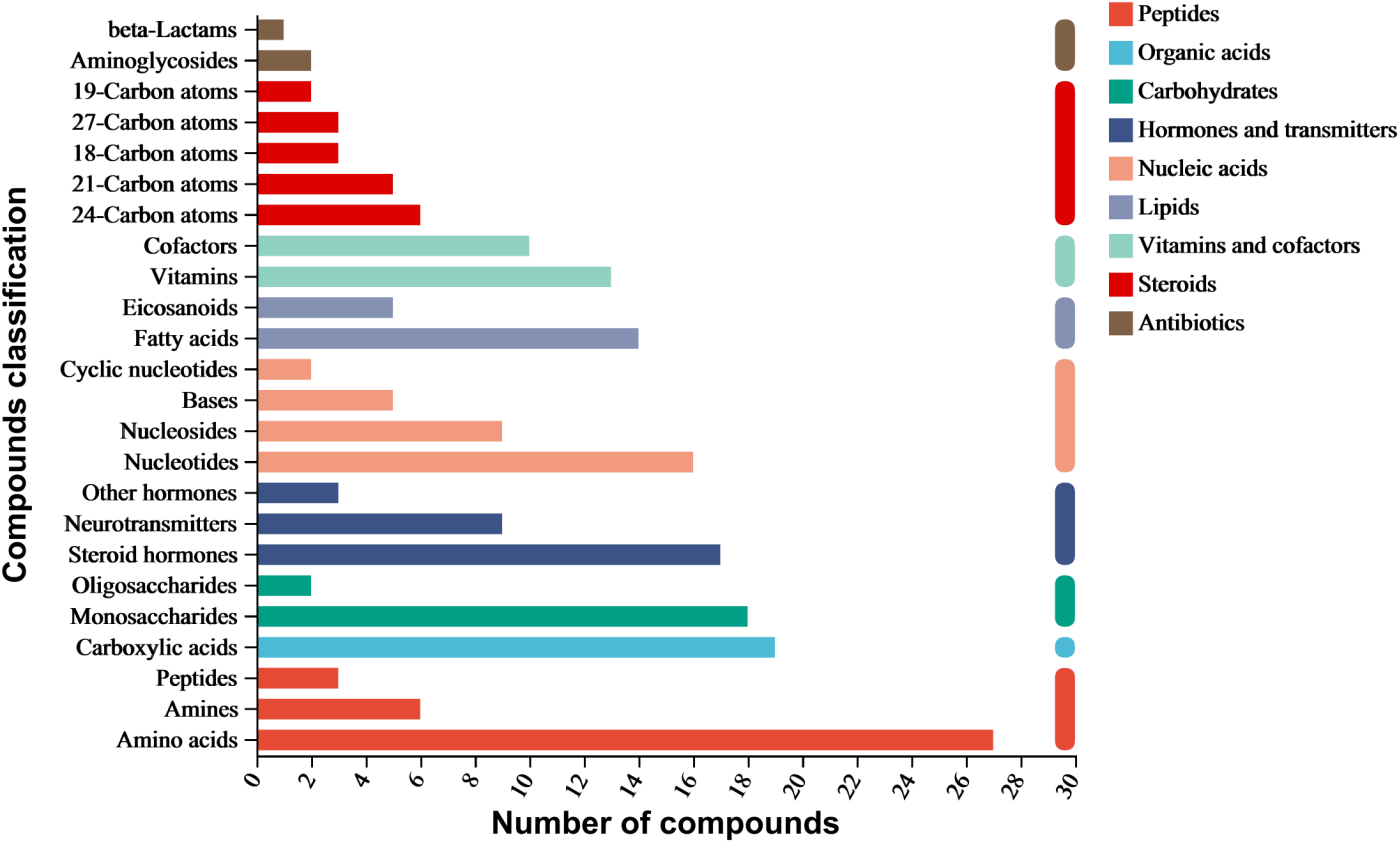
Compositional analysis of metabolites of *A. alternata* using KEGG database annotation.

The ethyl acetate extract of *A. alternata* was analyzed using LC-MS/MS, identifying 4036 metabolites categorized into nine classes: organic acids, lipids, carbohydrates, nucleic acids, peptides, vitamins and cofactors, steroids, hormones and transmitters, and antibiotics. Amino acids had the highest proportion, followed by carboxylic acids, monosaccharides, steroid hormones, nucleotides, fatty acids, vitamins, cofactors, neurotransmitters, nucleosides, and eicosanoids. In addition, aminoglycosides, beta-lactams, quinolones, and other antibiotics were also present (**Figure 3**).

Six antibiotics were detected in positive ion mode: cephalosporin C, cyclohexanimide, griseofulvin, mitomycin, nalidixic acid, and streptomycin. Additionally, seven antibiotics related to antimicrobial activity were detected in negative ion mode: ampicillin, chloramphenicol, fusiform acid, geldermycin, neomycin, penicillin G, and grandiomycin. These antibiotics mainly belong to beta-lactams, polyketides and nonribosomal peptides, quinolones, aminoglycosides, and other protein families (**Table 4**).

**Table 4:** Major antibiotics present in the metabolites of *A. alternate*.

ID	Metabolite	Category	Formula
pos_704	cephalosporin C	beta-lactams	C_16_H_21_N_3_O_8_S
pos_776	cyclohexanimide	others	C_15_H_23_NO_4_
pos_1056	griseofulvin	polyketides and nonribosomal peptides	C_17_H_17_ClO_6_
pos_1316	mitomycin	others	C_15_H_18_N_4_O_5_
pos_1412	nalidixic acid	quinolones	C_12_H_12_N_2_O_3_
pos_1855	streptomycin	aminoglycosides	C_21_H_39_N_7_O_12_
neg_391	ampicillin	beta-lactams	C_16_H_19_N_3_O_4_S
neg_495	chloramphenicol	others	C_11_H_12_Cl_2_N_2_O_5_
neg_710	fusiform acid	others	C_31_H_48_O_6_
neg_728	geldermycin	polyketides and nonribosomal peptides	C_29_H_40_N_2_O_9_
neg_1135	neomycin	aminoglycosides	C_23_H_46_N_6_O_13_
neg_1293	penicillin G	beta-lactams	C_16_H_18_N_2_O_4_S
neg_1419	grandiomycin	aminoglycosides	C_14_H_24_N_2_O_7_

**Figure 4:**
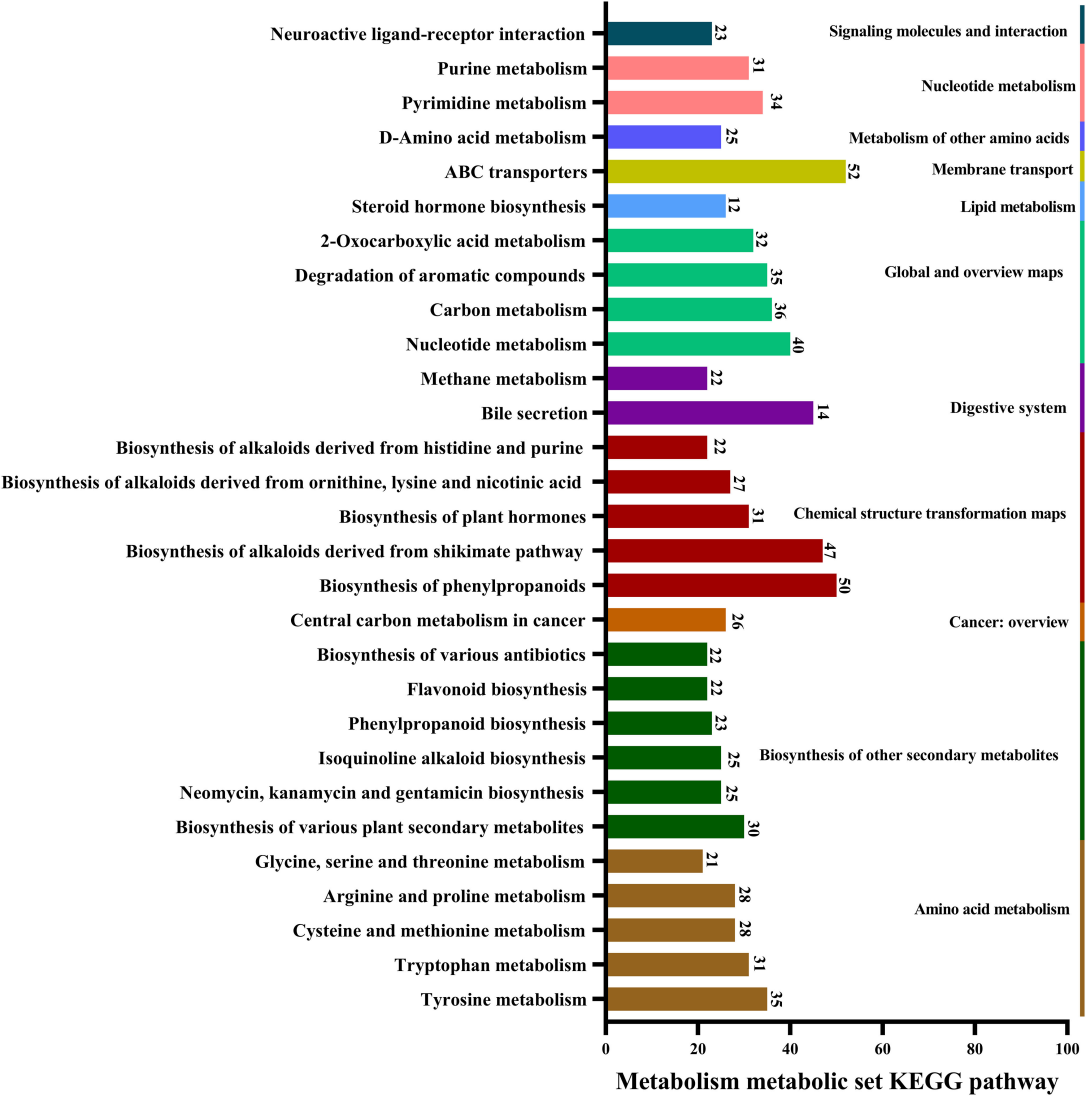
Major pathways involved in the metabolism of *A. alternata*, revealed by KEGG database annotations.

Metabolic pathways involving *A. alternata* metabolites were mainly related to ABC transporters, biosynthesis of phenylpropanoids, alkaloids derived from the shikimate pathway, nucleotide metabolism, carbon metabolism, tyrosine metabolism, pyrimidine metabolism, and degradation of aromatic compounds (**Figure 4**).

## 4 Discussion

Ascomycota and Basidiomycota were the dominant phyla of endophytic fungi found in the pedicel of *C. reticulata*. Previous studies have shown that in *C. oleifera*, Ascomycota is the most abundant phylum across all tissues, followed by Basidiomycota (Ercolani, 1991). Similarly, most plants also have Ascomycota as the dominant endophytic phylum, with Basidiomycota as the second most common, such as in *Aechmea*, *Fagopyrum tataricum*, and *Lunasia amara* (Rajamanikyam et al., 2017; Istikorini and Hartoyo, 2019; Leroy et al., 2019; Chen et al., 2024). However, most fungal species in *C. reticulata* pedicel were only classified to the order level, indicating the potential for discovering new species. Additionally, significant differences in the abundance of endophytic fungi at the family level indicate a diverse distribution of species in *C. reticulata*. No clear patterns were found in the dominant fungal families in *C. reticulata* pedicel compared to other *Camellia* species, possibly due to differences in the plant’s growth environment, which significantly influences endophytic fungal diversity.

Endophytic fungi are widely existed in plant tissues and an increasing number of these fungi have been isolated and identified (Hardoim et al., 2015). Their metabolites can effectively control diseases in agricultural and forestry crop (Abbasi et al., 2002). For instance, *Penicillium vinaceum*, isolated from *Crocus sativus*, secretes quinazoline alkaloids and exhibits significant antimicrobial activity against various pathogens (Zheng et al., 2012). Similarly, *Phomopsis cassiae*, isolated from *Cassia spectabilis*, produces five different sesquiterpenes and demonstrates strong antimicrobial activity against two plant-pathogenic bacteria (Silva et al., 2006). Nectar is rich in various nutrients, and yeast is commonly found within it. Yeast can significantly alter the composition and concentration of sugars and amino acids in nectar, accelerate flower aging, reduce attractiveness to pollinators, and impact the reproductive fitness of cross-pollinated plants (Herrera et al., 2008; Canto and Herrera, 2012). Given the antibacterial properties of endophytic fungi, they may assist nectar in antagonizing yeast proliferation. In this study, numerous endophytic fungi with inhibitory effects on yeasts have been screened from the *C. reticulata* pedicel, opening avenues for further research into the role of these endophytes in maintaining nectar stability. However, although research has shown that endophytes can change their colonization sites through rhizosphere competence and motility (Hardoim et al., 2015), the hypothesis that endophytic fungi within the pedicels could migrate to the nectar and influence its composition remains unconfirmed.

Endophytic fungi with antagonistic activity against nectar yeasts, isolated from the pedicel of *C. reticulata* under laboratory conditions, belong to Ascomycota phylum. Numerous species from the genera *Trichoderma*, *Penicillium*, and *Fusarium* are recognized as effective biocontrol agents. Fungi with antagonistic properties identified in previous studies predominantly belong to the Ascomycota, aligning with the findings of this study (Arnold and Lutzoni, 2007). The genus *Trichoderma* is a highly competitive biocontrol agent, with extensive research and applications documented (Peng et al., 2021). *Trichoderma* sp. protects several crops from diseases (Youssef et al., 2016; Swain et al., 2018). *Penicillium*, another endophytic genus, produces various antimicrobial compounds that have been used in crop disease and nematode control (Chen et al., 2022). Su et al. (2010) reported antibacterial activity in the culture filtrate of *Aspergillus* endophytes from tea trees, effective against *Camellia* brown spot disease. Furthermore, this study identified two antagonistic endophytic fungi only at the genus level, highlighting the potential for new species discovery in *C. reticulata*.

The identified *A. alternata* exhibited superior inhibition of nectar yeasts, affecting *M. reukaufii*, *C. laurentii*, and *R. glutinis*. Secondary metabolites from the genus *Alternaria* include pyrones, quinones, alkaloids, steroids, and terpenes, all of which demonstrate significant antimicrobial activity (Tantry et al., 2018; Lee et al., 2019; Chen et al., 2020). Specifically, capsaicin from *A. alternata* has been shown to exert antimicrobial effects against *Staphylococcus aureus*, *Escherichia coli*, and *Aspergillus niger* (Devari et al., 2014). Additionally, naphthalene and pyrone compounds from marine-derived *A. alternata* inhibit *Candida albicans* and *Fusarium oxysporum* (Shaaban et al., 2012). Alternaramide, a cyclic peptide from marine-derived *A. alternata*, is effective against *Bacillus subtilis* and *Staphylococcus aureus* (Kim et al., 2009). The identified metabolites in this study include antibiotics with antimicrobial properties, such as chloramphenicol, penicillin G, grandiomycin, and cephalosporin C, thus expanding the known data on *A. alternata* metabolites. Moreover, key metabolic pathways in *A. alternata* encompass the biosynthesis of plant secondary metabolites, phenylpropanoids, amino acids, antibiotics, and alkaloids. This analysis provides a basis for further research into its applications and mechanisms.

Importantly, nectar generally contains antimicrobial substances that are transported through the phloem and xylem during the nectar secretion process, enhancing pollination services and safeguarding rewards for flowers and/or pollinators (Adler, 2000; Stevenson et al., 2017). The nectar of *C. reticulata* is abundant and it depends on pollinators for fertilization. Endophytic fungi colonize the pedicels and produce a wealth of secondary metabolites, including various antibiotics. Under laboratory conditions, some of these fungi have demonstrated promising inhibitory effects against nectar yeasts. Our research findings provide insights into potential strategies that cross-pollinated plants may employ to cope with yeast stress, regulate nectar stability, and maintain reproductive fitness. However, further research is needed to determine whether metabolites secreted by endophytic fungi are transported from the phloem sieve tubes to the nectar to exert their effects, along with the underlying mechanisms and potential applications in antagonistic nectar yeasts.

## 5 Conclusion

Endophytic fungal diversity in pedicels of *C. reticulata* was high, primarily comprising Ascomycota and Basidiomycota. Twenty-seven isolates of endophytic fungi that exhibited antagonistic activity against three types of nectar yeasts were isolated and cultured. These isolates exhibited diverse morphologies and were identified into genera such as *Trichoderma*, *Fusarium*, and *Alternaria*. Among them, isolate D23, identified as *A. alternata*, demonstrated the highest antimicrobial potential. Its metabolites included antibiotics like penicillin G, grandiomycin, and cephalosporin C, and it participated in metabolic pathways related to phenylpropanoid and alkaloid biosynthesis. This research provided insights into the antagonistic effects of endophytic fungal metabolites against nectar yeasts in *C. reticulata* pedicels, emphasizing the importance of endophytes in assisting nectar maintain stability and support reproductive fitness in cross-pollinating plants.

## Data Availability

The original contributions presented in the study are included in the article/supplementary material. Further inquiries can be directed to the corresponding authors.

## References

[B1] Abbasi P. A. Al-Dahmani J. Sahin F. Hoitink H. A. J. Miller S. A. (2002). Effect of compost amendments on disease severity and yield of tomato in conventional and organic production systems. Plant Dis. 86, 156–161. doi: 10.1094/pdis.2002.86.2.156 30823313

[B2] Adler L. S. (2000). The ecological significance of toxic nectar. Oikos 91, 409–420. doi: 10.1034/j.1600-0706.2000.910301.x

[B3] Álvarez-Pérez S. Lievens B. Jacquemyn H. Herrera C. M. (2013). *Acinetobacter nectaris* sp. nov. and Acinetobacter boissieri sp. nov., isolated from floral nectar of wild Mediterranean insect-pollinated plants. Int. J. Systematic Evolutionary Microbiol. 63, 1532–1539. doi: 10.1099/ijs.0.043489-0 22904213

[B4] Arnold A. E. Lutzoni F. (2007). Diversity and host range of foliar fungal endophytes: are tropical leaves biodiversity hotspots? Ecology 88, 541–549. doi: 10.1890/05-1459 17503580

[B5] Brysch-Herzberg M. (2004). Ecology of yeasts in plant–bumblebee mutualism in Central Europe. FEMS Microbiol. Ecol. 50, 87–100. doi: 10.1016/j.femsec.2004.06.003 19712367

[B6] Canto A. Herrera C. M. (2012). Micro-organisms behind the pollination scenes: microbial imprint on floral nectar sugar variation in a tropical plant community. Ann. Bot. 110, 1173–1183. doi: 10.1093/aob/mcs183 22915578 PMC3478047

[B7] Cardoso V. M. Campos F. F. Santos A. R. O. Ottoni M. H. F. Rosa C. A. Almeida V. G.. (2020). Biotechnological applications of the medicinal plant *Pseudobrickellia brasiliensis* and its isolated endophytic bacteria. J. Appl. Microbiol. 129, 926–934. doi: 10.1111/jam.14666 32298521

[B8] Chen S. Deng Y. Yan C. Wu Z. Guo H. Liu L.. (2020). Secondary metabolites with nitric oxide inhibition from marine-derived fungus Alternaria sp. 5102. Mar. Drugs 18, 426. doi: 10.3390/md18080426 32823987 PMC7460390

[B9] Chen M. Ding Z. Zhou M. Shang Y. Li C. Li Q.. (2024). The diversity of endophytic fungi in Tartary buckwheat (*Fagopyrum tataricum*) and its correlation with flavonoids and phenotypic traits. Front. Microbiol. 15. doi: 10.3389/fmicb.2024.1360988 PMC10979544 38559356

[B10] Chen S. Tian D. Wei J. Li C. Ma Y. Gou X.. (2022). Citrinin derivatives from *Penicillium Citrinum* Y34 that inhibit α-Glucosidase and ATP-Citrate lyase. Front. Mar. Sci. 9. doi: 10.3389/fmars.2022.961356

[B11] Compant S. Duffy B. Nowak J. Clément C. Barka E. A. (2005). Use of plant growth-promoting bacteria for biocontrol of plant diseases: principles, mechanisms of action, and future prospects. Appl. Environ. Microbiol. 71, 4951–4959. doi: 10.1128/AEM.71.9.4951-4959.2005 16151072 PMC1214602

[B12] Devari S. Jaglan S. Kumar M. Deshidi R. Guru S. Bhushan S.. (2014). Capsaicin production by *Alternaria alternata*, an endophytic fungus from *Capsicum annum*; LC–ESI–MS/MS analysis. Phytochemistry 98, 183–189. doi: 10.1016/j.phytochem.2013.12.001 24378219

[B13] Ercolani G. L. (1991). Distribution of epiphytic bacteria on olive leaves and the influence of leaf age and sampling time. Microbial Ecol. 21, 35–48. doi: 10.1007/BF02539143 24194200

[B14] Feng G. (1980). Species and Utilization of *Camellia* in Yunnan (Kunming: Yunnan people’s Publishing House).

[B15] Gardes M. Bruns T. D. (1993). ITS primers with enhanced specificity for basidiomycetes - application to the identification of mycorrhizae and rusts. Mol. Ecol. 2, 113–118. doi: 10.1111/j.1365-294X.1993.tb00005.x 8180733

[B16] González-Mas N. Cuenca-Medina M. García-Mozo H. Muñoz-Redondo J. M. Moreno-Rojas J. M. Padilla-Álvarez F.. (2023). Endophytic Beauveria bassiana modifies flowering phenology, floral volatile profile and pollinator behaviour in melon. Entomologia Generalis 43, 961–969. doi: 10.1127/entomologia/2023/1991

[B17] Hardoim P. R. Overbeek L. S. V. Berg G. Pirttilä A. M. Compant S. Campisano A.. (2015). The hidden world within plants: Ecological and evolutionary considerations for defining functioning of microbial endophytes. Microbiol. Mol. Biol. Rev. 79, 293–320. doi: 10.1128/MMBR.00050-14 26136581 PMC4488371

[B18] Herrera C. M. de Vega C. Canto A. Pozo M. I. (2009). Yeasts in floral nectar: a quantitative survey. Ann. Bot. 103, 1415–1423. doi: 10.1093/aob/mcp026 19208669 PMC2701759

[B19] Herrera C. M. García I. M. Pérez R. (2008). Invisible floral larcenies: microbial communities degrade floral nectar of bumble bee-pollinated plants. Ecology 89, 2369–2376. doi: 10.1890/08-0241.1 18831156

[B20] Herrera C. M. Pozo M. I. Bazaga P. (2011). Clonality, genetic diversity and support for the diversifying selection hypothesis in natural populations of a flower-living yeast. Mol. Ecol. 20, 4395–4407. doi: 10.1111/j.1365-294X.2011.05217.x 21851437

[B21] Hiremani N. S. Verma P. Gawande S. P. Sain S. K. Nagrale D. T. Salunkhe V. N.. (2020). Antagonistic potential and phylogeny of culturable endophytic fungi isolated from desi cotton (*Gossypium arboreum* L.). South Afr. J. Bot. 134, 329–335. doi: 10.1016/j.sajb.2020.03.008

[B22] Istikorini Y. Hartoyo A. P. P. (2019). The diversity of endophytic fungi in kemaitan (*Lunasia amara* blanco). IOP Conf. Series: Earth Environ. Sci. 394, 12016. doi: 10.1088/1755-1315/394/1/012016

[B23] Jacquemyn H. Lenaerts M. Tyteca D. Lievens B. (2013). Microbial diversity in the floral nectar of seven *Epipactis* (Orchidaceae) species. MicrobiologyOpen 2, 644–658. doi: 10.1002/mbo3.103 23836678 PMC3948608

[B24] Kim M. Y. Sohn J. H. Ahn J. S. Oh H. (2009). Alternaramide, a cyclic depsipeptide from the marine-derived fungus Alternaria sp. SF-5016. J. Natural Products 72, 2065–2068. doi: 10.1021/np900464p 19943624

[B25] Lee C. Li W. Bang S. Lee S. J. Kang N. Kim S.. (2019). Secondary metabolites of the endophytic fungus *Alternaria alternata* JS0515 isolated from *Vitex rotundifolia* and their effects on pyruvate dehydrogenase activity. Molecules. 24, 4450. doi: 10.3390/molecules24244450 31817301 PMC6943735

[B26] Leroy C. Maes A. Q. Louisanna E. Séjalon-Delmas N. (2019). How significant are endophytic fungi in bromeliad seeds and seedlings? Effects on germination, survival and performance of two epiphytic plant species. Fungal Ecol. 39, 296–306. doi: 10.1016/j.funeco.2019.01.004

[B27] Peay K. G. Belisle M. Fukami T. (2012). Phylogenetic relatedness predicts priority effects in nectar yeast communities. Proc. R. Soc. B: Biol. Sci. 279, 749–758. doi: 10.1098/rspb.2011.1230 PMC3248732 21775330

[B28] Peng K. C. Lin C. C. Liao C. F. Yu H. C. Lo C. T. Yang H. H.. (2021). Expression of L-amino acid oxidase of *Trichoderma harzianum* in tobacco confers resistance to *Sclerotinia sclerotiorum* and *Botrytis cinerea* . Plant Sci. 303, 110772. doi: 10.1016/j.plantsci.2020.110772 33487356

[B29] Philippot L. Raaijmakers J. M. Lemanceau P. van der Putten W. H. (2013). Going back to the roots: the microbial ecology of the rhizosphere. Nat. Rev. Microbiol. 11, 789–799. doi: 10.1038/nrmicro3109 24056930

[B30] Pieterse C. M. J. Zamioudis C. Berendsen R. L. Weller D. M. Wees S. C. M. V. Bakker P. A. H. M. (2014). Induced systemic resistance by beneficial microbes. Annu. Rev. Phytopathol. 52, 347–375. doi: 10.1146/annurev-phyto-082712-102340 24906124

[B31] Pozo M. I. Lachance M. A. Herrera C. M. (2012). Nectar yeasts of two southern Spanish plants: the roles of immigration and physiological traits in community assembly. FEMS Microbiol. Ecol. 80, 281–293. doi: 10.1111/j.1574-6941.2011.01286.x 22224447

[B32] Pusey P. L. (1999). Effect of nectar on microbial antagonists evaluated for use in control of fire blight of pome fruits. Phytopathology 89, 39–46. doi: 10.1094/phyto.1999.89.1.39 18944801

[B33] Qin S. Chen K. Zhang W. Xiang X. Zuo Z. Guo C.. (2023). Phylogenomic insights into the reticulate evolution of *Camellia* sect. *Paracamellia* Sealy (Theaceae). J. Systematics Evol. 62, 38–54. doi: 10.1111/jse.12948

[B34] Rajamanikyam M. Vadlapudi V. Upadhyayula S. M. (2017). Endophytic fungi as novel resources of natural therapeutics. Braz. Arch. Biol. Technol. 60, e17160542. doi: 10.1590/1678-4324-2017160542

[B35] Richardson S. N. Nsiama T. K. Walker A. K. McMullin D. R. Miller J. D. (2015). Antimicrobial dihydrobenzofurans and xanthenes from a foliar endophyte of *Pinus strobus* . Phytochemistry 117, 436–443. doi: 10.1016/j.phytochem.2015.07.009 26189049

[B36] Schaeffer R. N. Mei Y. Z. Andicoechea J. Manson J. S. Irwin R. E. (2017). Consequences of a nectar yeast for pollinator preference and performance. Funct. Ecol. 31, 613–621. doi: 10.1111/1365-2435.12762

[B37] Shaaban M. Shaaban K. A. Abdel-Aziz M. S. (2012). Seven naphtho-γ-pyrones from the marine-derived fungus *Alternaria alternata*: structure elucidation and biological properties. Organic medicinal Chem. Lett. 2, 1–8. doi: 10.1186/2191-2858-2-6 PMC3350997 22377027

[B38] Silva G. H. Teles H. L. Zanardi L. M. Marx Young M. C. Eberlin M. N. Hadad R.. (2006). Cadinane sesquiterpenoids of *Phomopsis cassiae*, an endophytic fungus associated with *Cassia spectabilis* (Leguminosae). Phytochemistry 67, 1964–1969. doi: 10.1016/j.phytochem.2006.06.004 16857221

[B39] Stevenson P. C. Nicolson S. W. Wright G. A. (2017). Plant secondary metabolites in nectar: impacts on pollinators and ecological functions. Funct. Ecol. 31, 65–75. doi: 10.1111/1365-2435.12761

[B40] Stinson M. Ezra D. Hess W. M. Sears J. Strobel G. (2003). An endophytic Gliocladium sp. of *Eucryphia cordifolia* producing selective volatile antimicrobial compounds. Plant Sci. 165, 913–922. doi: 10.1016/S0168-9452(03)00299-1

[B41] Su J. Wang G. Yang M. (2010). Mixed culture of endophytic fungi isolated from *Camellia* sinensis enhancing the antagonistic on plant pathogenic fungi. Mycosystema 29, 753–759. doi: 10.13346/j.mycosystema.2010.05.013

[B42] Swain H. Adak T. Mukherjee A. K. Mukherjee P. K. Bhattacharyya P. Behera S.. (2018). Novel *Trichoderma* strains isolated from tree barks as potential biocontrol agents and biofertilizers for direct seeded rice. Microbiological Res. 214, 83–90. doi: 10.1016/j.micres.2018.05.015 30031485

[B43] Tantry M. A. Idris A. S. Williamson J. S. Shafi T. Dar J. S. Malik T. A.. (2018). Perylenequinones from an endophytic Alternaria sp. of *Pinus ponderosa* . Heliyon 4, e01046. doi: 10.1016/j.heliyon.2018.e01046 30603688 PMC6304448

[B44] Wang H. Liu Z. Duan F. Chen Y. Qiu K. Xiong Q.. (2023). Isolation, identification, and antibacterial evaluation of endophytic fungi from Gannan navel orange. Front. Microbiol. 14, 1172629. doi: 10.3389/fmicb.2023.1172629 37396354 PMC10307966

[B45] White T. J. Bruns T. D. Lee S. B. Taylor J. W. (1990). “Amplification and direct sequencing of fungal ribosomal RNA genes for phylogenetics,” in PCR Protocols: a guide to methods and applications. Eds. Innis M. A. Gelfand D. H. Sninsky J. J. White T. J. (Academic Press, San Diego), 315–322.

[B46] Youssef S. A. Tartoura K. A. Abdelraouf G. A. (2016). Evaluation of *Trichoderma harzianum* and *Serratia proteamaculans* effect on disease suppression, stimulation of ROS-scavenging enzymes and improving tomato growth infected by *Rhizoctonia solani* . Biol. Control 100, 79–86. doi: 10.1016/j.biocontrol.2016.06.001

[B47] Zhang H. Song Y. Tan R. (2006). Biology and chemistry of endophytes. Natural Product Rep. 23, 753–771. doi: 10.1039/B609472B 17003908

[B48] Zheng C. Li L. Zou J. Han T. Qin L. (2012). Identification of a quinazoline alkaloid produced by *Penicillium vinaceum*, an endophytic fungus from *Crocus sativus* . Pharm. Biol. 50, 129–133. doi: 10.3109/13880209.2011.569726 21517707

[B49] Zhou H. (2015). Concise botany tutorial (Wuhan: Central China Normal University Press).

